# Accuracy and precision of zero-echo-time, single- and multi-atlas attenuation correction for dynamic [^11^C]PE2I PET-MR brain imaging

**DOI:** 10.1186/s40658-020-00347-2

**Published:** 2020-12-28

**Authors:** João M. Sousa, Lieuwe Appel, Inés Merida, Rolf A. Heckemann, Nicolas Costes, Mathias Engström, Stergios Papadimitriou, Dag Nyholm, Håkan Ahlström, Alexander Hammers, Mark Lubberink

**Affiliations:** 1 Department of Surgical Sciences, Uppsala University, Uppsala, Sweden; 2 Medical Imaging Centre, Uppsala University Hospital, Uppsala, Sweden; 3 CERMEP, Lyon, France; 4 Department of Radiation Physics, Sahlgrenska Academy, University of Gothenburg, Gothenburg, Sweden; 5 GE Healthcare, Waukesha, USA; 6 Department of Neurology, Uppsala University Hospital, Uppsala, Sweden; 7 Department of Neurosciences, Uppsala University, Uppsala, Sweden; 8 King’s College London & Guy’s and St Thomas’ PET Centre, King’s College, London, UK; 9 Medical Physics, Uppsala University Hospital, Uppsala, Sweden

**Keywords:** MRAC, ZTE, Atlas, MaxProb, Dopamine transporter, Binding potential, rCBF

## Abstract

**Background:**

A valid photon attenuation correction (AC) method is instrumental for obtaining quantitatively correct PET images. Integrated PET/MR systems provide no direct information on attenuation, and novel methods for MR-based AC (MRAC) are still under investigation. Evaluations of various AC methods have mainly focused on static brain PET acquisitions. In this study, we determined the validity of three MRAC methods in a dynamic PET/MR study of the brain.

**Methods:**

Nine participants underwent dynamic brain PET/MR scanning using the dopamine transporter radioligand [^11^C]PE2I. Three MRAC methods were evaluated: single-atlas (Atlas), multi-atlas (MaxProb) and zero-echo-time (ZTE). The ^68^Ge-transmission data from a previous stand-alone PET scan was used as reference method. Parametric relative delivery (R_1_) images and binding potential (BP_ND_) maps were generated using cerebellar grey matter as reference region. Evaluation was based on bias in MRAC maps, accuracy and precision of [^11^C]PE2I BP_ND_ and R_1_ estimates, and [^11^C]PE2I time-activity curves. BP_ND_ was examined for striatal regions and R_1_ in clusters of regions across the brain.

**Results:**

For BP_ND_, ZTE-MRAC showed the highest accuracy (bias < 2%) in striatal regions. Atlas-MRAC exhibited a significant bias in caudate nucleus (− 12%) while MaxProb-MRAC revealed a substantial, non-significant bias in the putamen (9%). R_1_ estimates had a marginal bias for all MRAC methods (− 1.0–3.2%). MaxProb-MRAC showed the largest intersubject variability for both R_1_ and BP_ND_. Standardized uptake values (SUV) of striatal regions displayed the strongest average bias for ZTE-MRAC (~ 10%), although constant over time and with the smallest intersubject variability. Atlas-MRAC had highest variation in bias over time (+10 to − 10%), followed by MaxProb-MRAC (+5 to − 5%), but MaxProb showed the lowest mean bias. For the cerebellum, MaxProb-MRAC showed the highest variability while bias was constant over time for Atlas- and ZTE-MRAC.

**Conclusions:**

Both Maxprob- and ZTE-MRAC performed better than Atlas-MRAC when using a ^68^Ge transmission scan as reference method. Overall, ZTE-MRAC showed the highest precision and accuracy in outcome parameters of dynamic [^11^C]PE2I PET analysis with use of kinetic modelling.

## Introduction

In positron emission tomography (PET), photon attenuation correction (AC) is a prerequisite for obtaining dependable images and quantifications. Photon attenuation is the largest correction required in PET imaging as photon attenuation is inhomogeneous across tissues [1]. The amount of attenuation depends on spatially varying electron density. PET AC routines therefore require knowledge about the spatial distribution of attenuation coefficients within the field of view (FOV) of the scanner, represented as an attenuation map whose voxel values denote linear attenuation coefficients [2–4].

Stand-alone PET systems are most often equipped with rotating ^68^Ge/^68^Ga transmission sources [5, 6]. This method is usually regarded as the gold standard for AC [7] as it measures 511 keV photon attenuation directly, however not without some drawbacks like noise and poor resolution. In PET/computed tomography (CT) systems, AC is based on low-dose CT scanning [8], with CT images converted from Hounsfield units to linear attenuation coefficients at 511 keV to obtain a suitable AC map for PET correction [9].

Integrated PET/magnetic resonance imaging (MRI) systems offer new opportunities and challenges. Neurological disorders are key applications with a long clinical tradition of combining functional information from nuclear medicine images with structural information from magnetic resonance imaging (MRI) to allow adequate quantification across the brain. However, MRI provides no direct information on electron density that can be used for AC [10, 11]. Instead, MRI can supply proton density, but its correlation with gamma photon attenuation is highly nonlinear: the bone, in particular, has low proton density and high electron density [12, 13]. Additional efforts are needed to determine which AC method can be implemented as a routine in an image reconstruction process.

Several approaches have been reported for MR-based attenuation correction (MRAC) of PET brain data. Initially, a Dixon-based approach was implemented for PET MRAC. This method is based on segmentation into tissue classes (i.e. air, lung, fat and soft tissue) to which fixed attenuation coefficients are assigned [14–16]. As the Dixon-based approach is primarily intended for whole-body PET-MR, the skull is not considered. As a consequence, this MRAC method is a source of a substantial bias in brain studies, especially for cortical regions [17–19]. In parallel, various atlas-based MRAC approaches were developed and evaluated. In this broad class of methods, a single atlas [20, 21] or multiple atlases [22, 23] are applied to incorporate the bone in the MRAC map. As no direct relationship exists between MRI and CT [3], various statistical methods have been applied to convert MR intensities to a pseudo-CT with Hounsfield units for discrimination of soft tissue, air and bone [24]. Generally, atlas-based MRAC methods improve accuracy by including the skull and seem to perform within acceptable quantitative limits [11, 25]. Most recently, segmentation of a zero-echo-time (ZTE) MRI sequence [12, 26, 27] has been suggested for MRAC. With some resemblance to ultrashort echo time (UTE) [28] and more recently RESOLUTE [29], ZTE was designed to obtain signal from cortical bone with both lower acoustic noise and reduced sensitivity to gradient fidelity artefacts [26, 30, 31]. As such, ZTE could be used to incorporate the bone in a MRAC map [10, 12, 26]. Several clinical evaluations demonstrated for static PET-data acquisitions that ZTE-based MRAC accomplished quantification with smaller errors than a single-atlas approach, both with CT-AC [10, 32–37] or ^68^Ge-AC as reference [38]. Another rapidly emerging field is the use of neural-networks to incorporate the bone in MRAC [39–42] but these approaches are not considered here.

Until now, evaluations of various MRAC methods have mainly focused on static brain PET, showing merely the tracer uptake or target-to-reference ratios. In an independent multi-centre study where eleven MRAC methods were applied to a large cohort data set of static fluorodeoxyglucose (FDG) PET/MR data, the average performance in PET tracer uptake was generally within ± 5% of CT-AC [25]. However, dynamic brain PET holds the compelling promise of in vivo functional imaging, i.e. following physiological processes over time, or obtaining quantitative measures by means of tracer kinetic modelling. Several recent studies have reported results for dynamic PET data using two scanners, MRAC methods and radiotracers as well as different kinetic modelling approaches depending on the tracer kinetics [43–46]. To date, no study has compared the multi-atlas method maximum probability algorithm (MaxProb) [43], favoured by Ladefoged et al. [25], the single-atlas method and ZTE using the gold standard ^68^Ge-AC as a reference, in a dynamic setting. MaxProb-MRAC builds on a database of brain MR/CT image pairs and an application of a novel maximum probability method. In a dynamic setting with the serotonergic 1a tracer [^18^F]MPPF, an acceptable amount of bias in the estimated binding potential (BP_ND_) was seen using CT-AC as reference.

In this study, we used ^11^C-labelled PE2I (N-(3-iodoprop-2E-enyl)-2β-carbomethoxy-3β-(4-methyl-phenyl) nortropane), a radiotracer with high affinity to dopamine transporters (DAT), especially in the striatum [47]. Kinetic modelling of dynamic [^11^C]PE2I imaging data is able to determine both BP_ND_, proportional to DAT availability, and relative delivery (R_1_) reflecting relative cerebral blood flow, from one single brain scan [48, 49]. These quantitative outcome measures are particularly important for the differential diagnosis in patients with Parkinsonism [50] and used in clinical routine at our centre. Despite the promising evaluation reports of novel MRAC methods, it is still unclear how these approaches might perform in a dynamic dataset with a specific dopamine tracer and use of a kinetic modelling approach.

The aim of this study was to determine the validity of three different MRAC methods in a setting with a dynamic [^11^C]PE2I PET brain scan on a PET-MR system, using ^68^Ge-transmission AC as a reference method. We evaluated (1) a single-atlas method (Atlas-MRAC) [11], (2) an MR sequence-based method (ZTE-MRAC) [12, 26, 27] and (3) a multiple-atlas method (MaxProb-MRAC) [43, 51] in a dynamic [^11^C]PE2I PET dataset acquired on an integrated digital time-of-flight PET-MR system. Individual transmission scans acquired on a stand-alone PET system were used as the reference method (^68^Ge-MRAC), directly measuring attenuation at 511 keV.

## Methods

### Participants and data acquisition

The participants and data acquisition have previously been described [38]. This study was approved by the Regional Board of Medical Ethics in Uppsala as well as the Radiation Ethics Committee of Uppsala University Hospital, and all participants consented in writing to take part in the study before inclusion. The study comprised nine patients with Parkinsonism (5 females, 4 males; median age 72 years, range 49–82). Previously, each participant had undergone a 10-min transmission scan using three rotating ^68^Ge rod sources on an ECAT Exact HR+ scanner (Siemens, Knoxville, USA), prior to injection of any radioactivity. Participants then underwent a dynamic brain PET scan using ^11^C-PE2I on a 3-T time-of-flight (TOF) PET/MR (SIGNA PET/MR, GE Healthcare, Waukesha, USA) system.

An 80-min PET scan was acquired in list mode, starting simultaneously with intravenous bolus administration of 5 MBq/kg [^11^C]PE2I using an infusion pump, and divided into 22 time frames of increasing duration (4 × 60s, 2 × 120 s, 4 × 180 s, 12 × 300 s). Relevant MR sequences were acquired during the first five minutes of the [^11^C]PE2I PET scan to avoid misalignment due to head movements. Three MR sequences were acquired during the [^11^C]PE2I PET scan: (1) a T1w 3D LAVA Flex that was later used for Atlas-MRAC (duration 18 s, 1 number of excitations (NEX), FOV 500 mm, slice thickness 5.2 mm, overlap 2.6 mm, matrix 256 × 256 and 5° flip angle); (2) a proton-density ZTE sequence (duration 153 s, 4 NEX, FOV 260 mm, slice thickness 1.4 mm, no slice gap, matrix 192 × 192, flip angle 0.8°); and (3) a 3D T1w brain volume imaging sequence that was later used for definition of regions of interest and as the target for MaxProb-MRAC registration (gradient-echo, duration 272 s, 1 NEX, FOV 250 mm, slice thickness 1 mm, matrix 256 × 256, flip angle 12°, TI 450 ms).

### Generation of MR attenuation correction maps

For each participant, the following MR attenuation correction maps (MRAC) were produced:

*Atlas-MRAC*—The single-atlas-based method [11, 20] consisted of three steps: (1) application of a Hessian matrix to enhance bone structures in the T1w image, (2) pseudo-CT generation by registration of the enhanced image to a head atlas based on CT scans of 50 participants, and (3) standard energy conversion and resampling of the pseudo-CT, resulting in an AC map with dimensions of 128 × 128 × 89 voxels (4.68 × 4.68 × 2.78 mm). This method was vendor-provided (software version MP26) as a standard application for our PET/MR system when the study was initiated.

*ZTE-MRAC*—In this procedure, an intensity equalization to a ZTE image was followed by logarithmic image rescaling to enhance bone tissue. Next, a mask was used to isolate the brain data, thus removing bed and coil information. A sequence of thresholding operations was then applied to the subject’s brain image to segment the bone and air regions by fitting a Gaussian to the main image histogram peaks. Remaining internal air compartments were further segmented by means of histogram thresholding. ZTE bone voxels are assigned continuous attenuation coefficients using a ZTE-intensity HU calibration curve as described in [10, 12, 26, 33]. Finally, the resulting MRAC image was co-registered and resampled to the subjects’ individual Atlas-MRAC by applying 6-parameter rigid-body registration creating the ZTE-MRAC map. This method is also a vendor-implemented process (GE PET Reconstruction Toolbox MP26), but during data acquisition still under development [12, 26, 27].

*MaxProb-MRAC*—This method utilized a database of 40 corresponding MR and CT images [43, 51]. Processing steps were executed using an in-house MATLAB pipeline (MATLAB R2017a, Mathworks Inc., Natick, MA, USA). Each MR image from the database was paired with the participant’s high-resolution T1w image, and masking was applied to both images to reduce extraneous image information. In the following registration steps, the masked MR image pair was aligned by means of affine registration followed by non-rigid registration using normalized mutual information as the similarity measure (register github.com/BioMedIA/MIRTK). Subsequently, the transformation matrices acquired from the non-rigid registration of MR images were applied to the corresponding CT images, yielding a database of co-registered MR/CT image pairs. In the next step, the co-registered CTs were segmented into three tissue classes defined by intensity thresholds (air: < − 500 HU, soft tissue: − 500–300 HU, bone: > 300 HU [20]. Each voxel tissue class of the target subject space was then assigned to a tissue class by majority voting of tissue class labels across the registered CT database. Finally, a voxel-wise pseudo-CT was generated by averaging CT Hounsfield values of atlases belonging to the majority class for the corresponding voxel. A bilinear energy conversion [52] was performed as the last step of attenuation map computation. The resulting MRAC image was rigidly co-registered and resampled to the participant’s individual Atlas-MRAC. Thereafter, the MaxProb-MRAC image was completed by adding, from the Atlas-MRAC map, the neck information missing due to differences in axial FOV between the images retrieved from the MR/CT database (216 mm) and the PET/MR scanner (250 mm).

^*68*^*Ge-AC*—Attenuation maps were reconstructed from the ^68^Ge transmission scan using ordered subset expectation maximisation (OSEM) with 6 iterations, 8 subsets and a 4 mm Hanning post-filter, dimensions 128 × 128 × 63 voxels (5.15 × 5.15 × 2.43 mm). Thereafter, a mask was applied to strip the AC map from the bed and head support as well as noise surrounding the head, followed by rigid co-registration and resampling to the participant’s individual Atlas-MRAC map. As the axial FOV differed between the stand-alone PET (155 mm) and PET/MR scanner (250 mm), the ^68^Ge-AC map was completed with the corresponding information concerning the neck and top of the head from the Atlas-MRAC map.

As a common step for all generated MRAC maps, coil and bed AC map templates were then incorporated, applying an in-house MATLAB pipeline (MATLAB R2017a, Mathworks Inc., Natick, MA, USA).

### Image reconstruction

The dynamic [^11^C]PE2I PET data were reconstructed using time-of-flight OSEM with 2 iterations, 28 subsets, 5 mm Gaussian post-filter, 128 × 128 reconstruction matrix and 300 mm FOV. MRAC was performed in four ways as described in the previous section. Further, all appropriate corrections for quantitative image reconstruction were applied.

### [11C]PE2I image analysis

The methodology and validation for voxel-level analysis of dynamic [^11^C]PE2I scans have been previously reported [48, 49].

Following this approach, the reconstructed [^11^C]PE2I PET images were realigned to correct for interframe patient movements using an early (0–3 min) [^11^C]PE2I summed image as reference using an in-house MATLAB 2018 script. Interframe motion was estimated in this way for the PET images corrected for AC using ^68^Ge-AC, and the same transformation were applied to the other three dynamic data sets. Subsequently, the same summed image was used for co-registration of the 3D T1w MRI scan based on a 6-parameter rigid transformation, to achieve positional alignment. Then, MR images were segmented into grey matter, white matter, and cerebrospinal fluid using Statistical Parametric Mapping (SPM12; Wellcome Trust Center for Neuroimaging, University College London, UK). Grey matter volumes of interest (VOI) were established on the T1w structural MR images using an automated probabilistic template as implemented in the PVElab software [53] for cortical and limbic regions. For striatal regions, MAPER [54], a technique optimized for segmentation of atrophic brains, was used to achieve a more accurate delineation. All VOIs were projected over the dynamic scans to generate time-activity curves (TACs).

Parametric images were generated from the [^11^C]PE2I scan using receptor parametric mapping (RPM) with cerebellar grey matter as a reference region [55]*.* RPM is an implementation of the simplified reference tissue model, SRTM [56], with a set of predefined basis functions to linearize the model and estimate voxel-wise R_1_ and BP_ND_ that can be applied to [^11^C]PE2I. The parametric [^11^C]PE2I BP_ND_ images demonstrate specific binding of [^11^C]PE2I to DAT directly proportional to DAT density (availability) and therefore mainly show the deep grey matter of the striatum. The parametric [^11^C]PE2I R_1_ images display relative cerebral blood flow, which reveals overall brain functional activity. This procedure was repeated for all four [^11^C]PE2I PET data sets. Finally, the grey matter VOIs on the co-registered MR images were projected on the various parametric [^11^C]PE2I R_1_ and BP_ND_ images from all four datasets.

### Volumes of interest

Quantification of BP_ND_ was evaluated separately for the caudate nucleus and putamen as well as for the whole dorsal striatum (volume-weighted average of the putamen and caudate). These regions normally have high DAT availability, but a pronounced reduction is often observed in persons with Parkinsonism [49].

Evaluation of the quantification of R_1_ was based on four clusters of VOIs across the brain: anterior cortical regions (cingulate, frontal gyrus), posterior cortical regions (occipital cortex, parietal cortex, somatosensory-motor cortex), dorsal striatal regions (caudate nucleus, putamen) and limbic regions (amygdala, hippocampus, hypothalamus, thalamus) [49]. In addition, whole-brain grey matter (WB) was determined as an overall measure.

### Evaluation of MRAC maps

All participants’ MRAC maps were converted to MNI space using SPM12. Mean MRAC images for all four methods were calculated, and bias compared to ^68^Ge-AC was calculated at the voxel level as follows:

1 $$ {Bias}_i={MRAC}_i-{}^{68} GeAC $$

where *i* refers to the three MRAC methods being compared to ^68^Ge-AC.

Attenuation maps were assessed on soft tissue and bone compartments. A large VOI was drawn over the ^68^Ge-AC map soft tissue and copied to the other AC methods. A bone mask was created by simple segmentation of the ZTE map followed by 2-pixel erosion to reduce the probability of coinciding with other tissues. The bone mask was then transferred to all other AC methods. Mean bias values were calculated for both soft tissue and bone.

### Evaluation of [11C]PE2I BPND and R1 estimates

[^11^C]PE2I BP_ND_ and R_1_ images for each subject were converted to MNI space using SPM12. Mean [^11^C]PE2I BP_ND_ and R_1_ images for all four methods were calculated, and bias (Eq. 1) compared to ^68^Ge was calculated at the voxel level for the defined VOIs.

For quantitative assessment, we used relative differences (% bias; see Eq. 2) in BP_ND_ and R_1_ as a measure of accuracy, while standard deviation of the bias was taken as a measure of precision.

2 $$ {Bias}_i\left(\%\right)=\frac{PET_{MRAC_i}-{PET}_{68 Ge- AC}}{PET_{68 Ge- AC}}\times 100\kern0.75em $$

with *i* referring to the three evaluated MRAC methods.

Correlation analysis (Spearman) and Deming regression were used to assess the degree of agreement between BP_ND_ and R_1_ values obtained using each of the evaluated MRAC methods and the reference method (^68^Ge-AC).

Statistical analysis was performed using GraphPad Prism 6 (GraphPad Software, La Jolla, CA, USA). Significant differences in bias (*p* < 0.05) between each evaluated MRAC method and ^68^Ge-AC as reference method were assessed using a Friedman test with post-hoc tests (comparison to ^68^Ge-AC) and Dunn’s correction.

### Evaluation of time-activity curves

Previous reports indicate that effects of attenuation correction on uptake over time can be inhomogeneous [29, 43, 44, 57] and may potentially affect the accuracy and precision of the parameters estimated by kinetic modelling. We therefore examined relative bias and its SD in [^11^C]PE2I standardized uptake values (SUV) over time. This was done for striatal regions with a high DAT density and the reference region (cerebellum) used after implementation of Atlas-, ZTE- and MaxProb-MRAC.

## Results

### MRAC maps

**Fig. 1:**
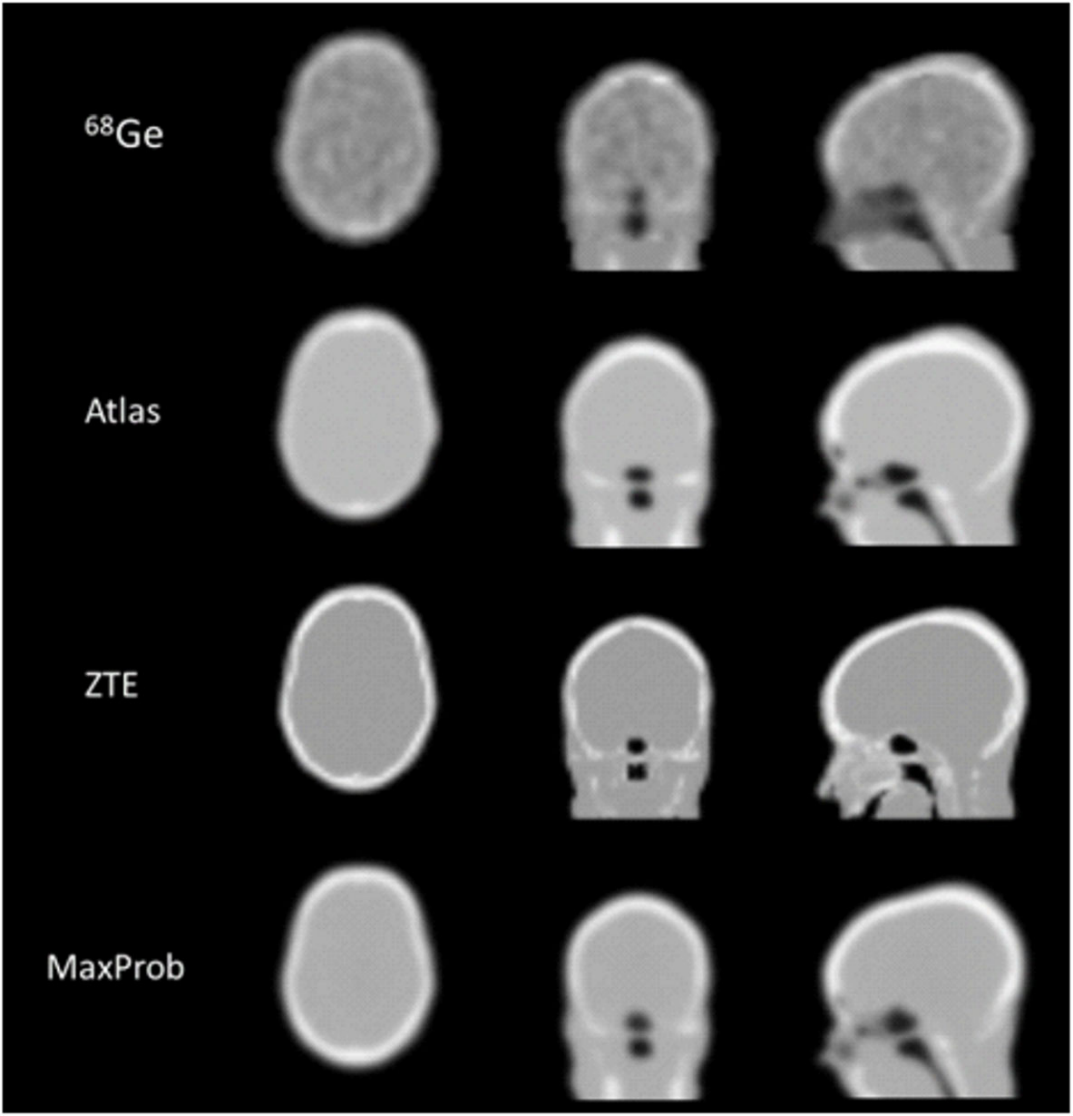
Typical MRAC maps-based on ^68^Ge, Atlas, ZTE and MaxProb approaches. Neck completion was required for ^68^Ge-AC and MaxProb-MRAC and was based on Atlas-MRAC

The MRAC maps for ^68^Ge, Atlas, ZTE and MaxProb of a representative subject are presented in Fig. 1. Each MRAC map exhibited distinct qualitative features mainly inherent to how the maps were generated. The ^68^Ge-AC map displayed images with heterogeneous signal in the soft tissue due to noise as a result of low-count statistics. The ZTE-MRAC map based on MRI methodology had the highest resolution, although the sinus regions are not well defined. The Atlas- and MaxProb-MRAC maps were qualitatively similar. Furthermore, ^68^Ge and MaxProb-MRAC maps were completed with Atlas-based information.

**Fig. 2:**
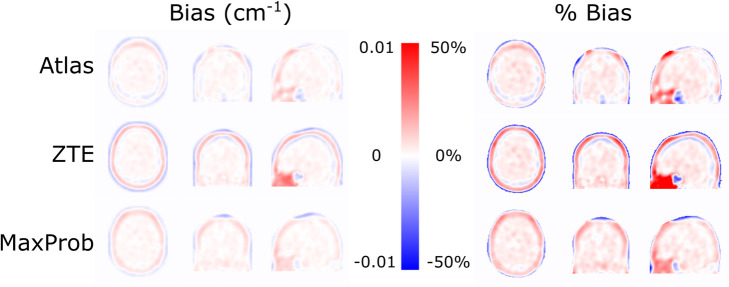
Mean absolute and relative bias across participants in Atlas-, ZTE- and MaxProb-MRAC maps. Positive bias in red and negative in blue

Figure 2 shows the mean bias across all participants in brain attenuation maps for the evaluated MRAC methods. In brain soft tissue, the smallest mean bias was found for ZTE-MRAC (2.9 · 10^−4^ cm^−1^) and MaxProb-MRAC (3.0 · 10^−4^ cm^−1^) closely followed by Atlas-MRAC (3.3 · 10^−4^ cm^−1^). In the bone, Atlas-MRAC yielded the lowest mean bias (5.6 · 10^−4^ cm^−1^) compared with an approximately two times higher bias for MaxProb-MRAC (13 · 10^−4^ cm^−1^) and three times higher bias for ZTE-MRAC (18 · 10^−4^ cm^−1^). In the sinus region, an evident bias was detected in all maps where the highest overestimation was noticed for ZTE-MRAC.

### Binding potential

**Fig. 3:**
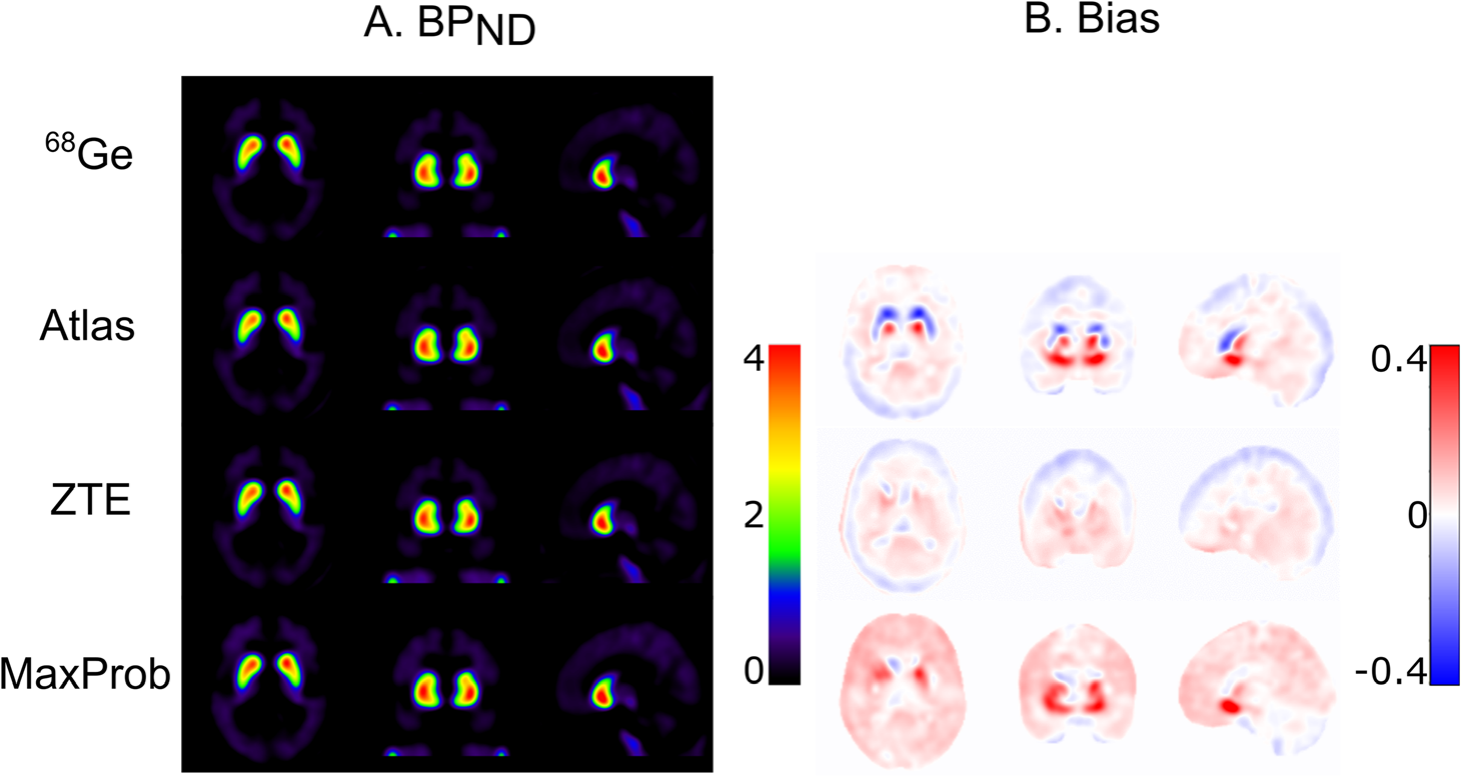
**a** Mean BP_ND_ images using ^68^Ge- and Atlas-, ZTE- and MaxProb-MRAC showing striatal DAT density. **b** Mean bias images of BP_ND_ illustrating voxel-wise differences in BP_ND_ between each MRAC method and ^68^Ge-MRAC

Mean parametric BP_ND_ images were similar for all MRAC methods (Fig. 3a). Mean bias images of BP_ND_ displayed an obvious negative bias in the striatum in case of Atlas-MRAC while these images pointed to a relatively small striatal bias for ZTE- and MaxProb-MRAC (Fig. 3b). Various bias patterns between MRAC methods were found for extrastriatal regions but this might also be due to low DAT density (see Fig. 3b).

**Fig. 4:**
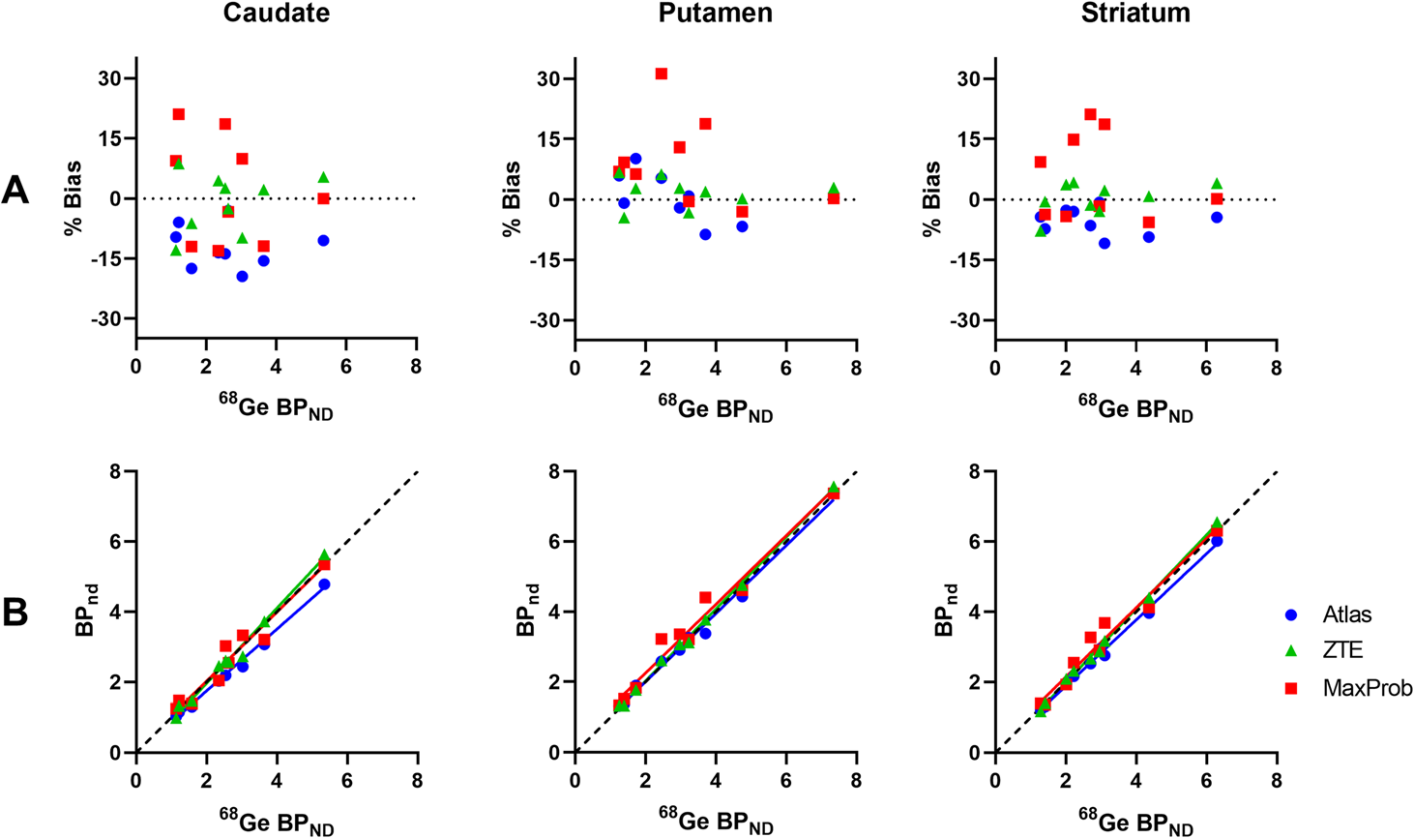
**a** Relative differences in BP_ND_ (% bias) plots of BP_ND_ when comparing Atlas-, ZTE- or MaxProb-MRAC to ^68^Ge-AC (gold standard) for striatal brain regions. **b** Relationship between BP_ND_ using various MRAC methods and ^68^Ge-AC as reference, where solid lines represent Deming regressions and dashed line identity

The relative bias of the striatal BP_ND_ estimates is presented in Fig. 4a. Atlas-MRAC resulted in a substantial negative relative bias for the caudate and the entire striatum, but only a small bias for the putamen. For ZTE, a minor relative bias in BP_ND_ with a low variability was observed for the putamen and striatum, whereas a greater variability between participants was noted for the caudate. For all regions, MaxProb showed considerable variability with both positive and negative bias percentages. There was no obvious relationship between the magnitude of the reference values (BP_ND_ based on ^68^Ge-AC) and bias percentages.

The Deming regression lines demonstrated a high degree of agreement for ZTE- and MaxProb-MRAC compared to the reference method in all brain regions (Fig. 4b). In contrast, the regression lines for Atlas-MRAC exhibited a deviation from the identity line, indicating a negative bias compared to the reference method.

Table 1 lists quantitative values related to accuracy (% bias) and precision (SD of % bias) for estimated BP_ND_ values as well as measures of consistency between evaluated and reference MRAC (correlations, regression parameters). ZTE-MRAC results showed little bias for all regions (< 2%) with a generally high precision. The highest bias was found for Atlas-MRAC in the caudate (approximately − 12%) which differed significantly from ^68^Ge-AC (*p* < 0.05). MaxProb also showed a relatively large mean bias in the putamen (about 9%) but did not deviate significantly from ^68^Ge-MRAC. MaxProb-MRAC showed the lowest precision in all regions. Only the bias obtained for the caudate with the Atlas approach was statistically significant.

**Table 1:** BP_ND_–mean % bias and precision (SD of % bias) as well as correlation coefficient (*r*) for striatal regions when comparing Atlas-, ZTE- and MaxProb-MRAC against ^*68*^Ge-AC. Additionally, the slope and intercept of Deming regression lines are given

Brain region	MRAC	% bias	SD	*r*	Slope	Intercept
CN	Atlas	− 12.09*	5.36	0.98	0.88	− 0.01
	ZTE	− 0.85	7.41	0.95	1.07	− 0.18
	MaxProb	2.11	13.24	0.98	0.98	0.06
PUT	Atlas	0.52	6.04	1.00	0.97	0.07
	ZTE	1.83	3.81	0.95	1.02	− 0.02
	MaxProb	9.19	10.78	1.00	0.98	0.29
STR	Atlas	− 5.43	3.31	0.98	0.94	0.00
	ZTE	0.30	3.95	0.97	1.05	− 0.11
	MaxProb	5.48	10.62	1.00	0.99	0.17

*CN* caudate nucleus, *PUT* putamen, *STR* striatum

*p value < 0.05. Friedman test with post-hoc tests (comparison to ^68^Ge-AC) and Dunn’s correction

In general, strong correlations with ^68^Ge-AC were established for all three evaluated MRAC- methods in all regions (*r* ≥ 0.95).

**Fig. 5:**
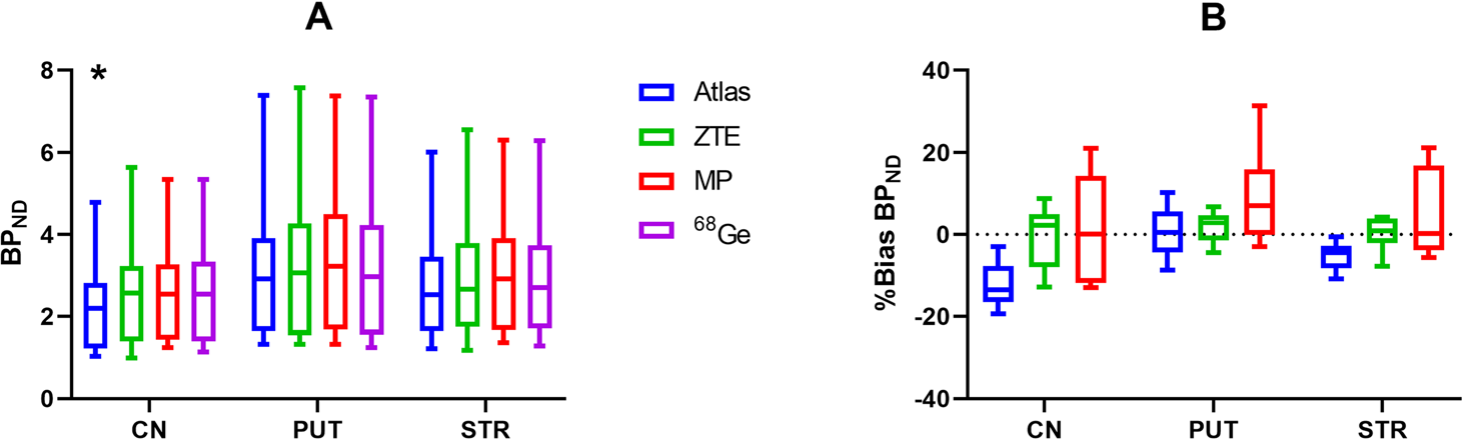
Boxplots for estimated BP_ND_ (**a**) and % bias (**b**) in BP_ND_ when comparing Atlas-, ZTE- or MaxProb-MRAC to ^*68*^Ge-AC for the caudate (CN), putamen (PUT), and entire striatum (STR). Bars and whiskers represent median and first and third quartile. * *p* value < 0.05

Estimated BP_ND_ values and relative bias for the evaluated MRAC methods are illustrated with boxplots in Fig. 5. The median BP_ND_ of the investigated brain regions differed only modestly within regions (Fig. 5a). The relative bias was low for ZTE-MRAC while MaxProb-MRAC showed the lowest precision indicated by the size of the boxes and bars (Fig. 5b).

### Relative delivery

**Fig. 6:**
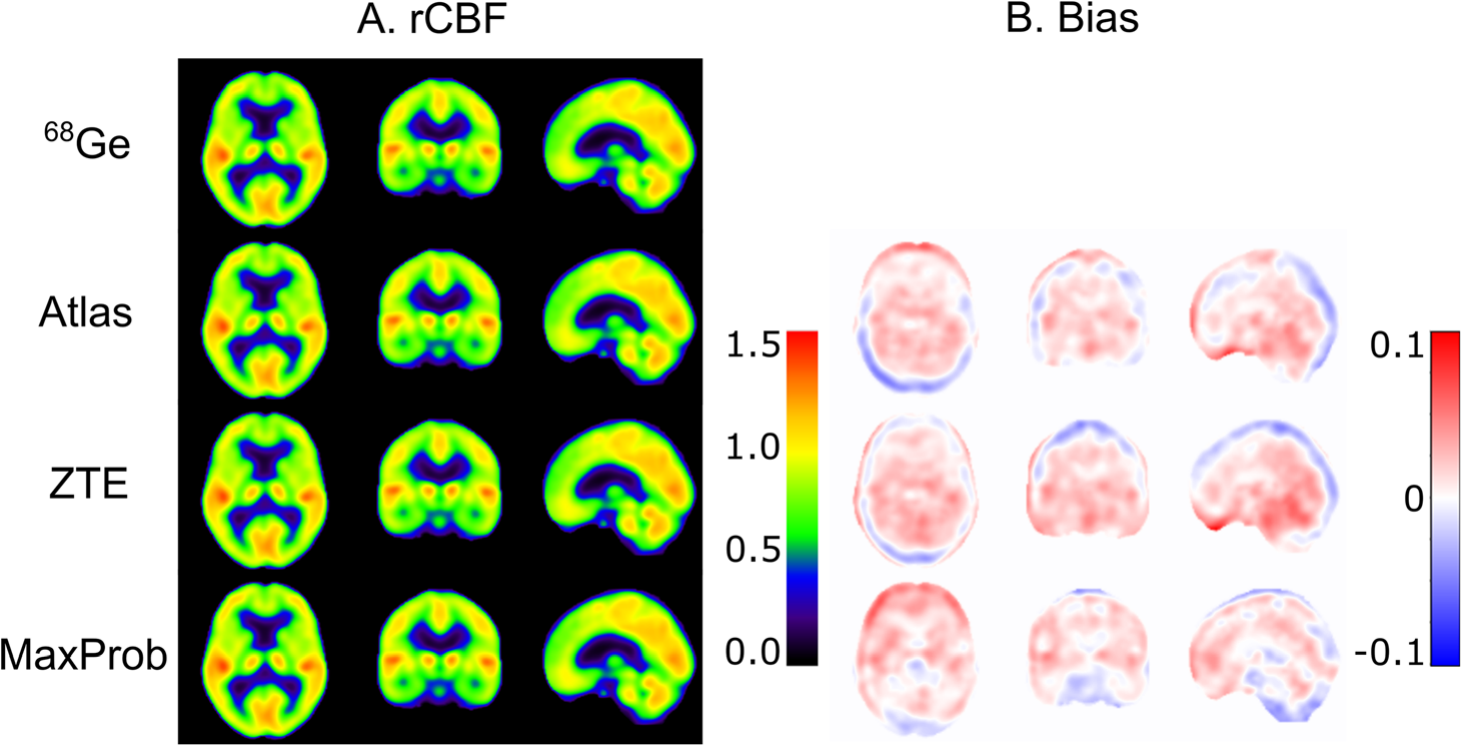
**a** Mean parametric R_1_ images using ^68^Ge- and Atlas-, ZTE- and MaxProb-MRAC. **b** Mean bias images of R_1_ illustrating voxel-wise differences in R_1_ between each MRAC method and ^68^Ge-AC

Mean parametric R_1_ images, representing relative cerebral blood flow and overall brain activity, are presented in Fig. 6a and show similar patterns for all four MRAC methods. Mean bias images of R_1_ indicated mostly a positive bias throughout the brain regardless of the MRAC method used (Fig. 6b). Further, these images pointed to a distinct positive bias in the anterior part of the brain for Atlas-MRAC and especially MaxProb-MRAC. Negative bias was evident in the posterior part of the brain for Atlas-MRAC and to a lesser degree for ZTE.

**Fig. 7:**
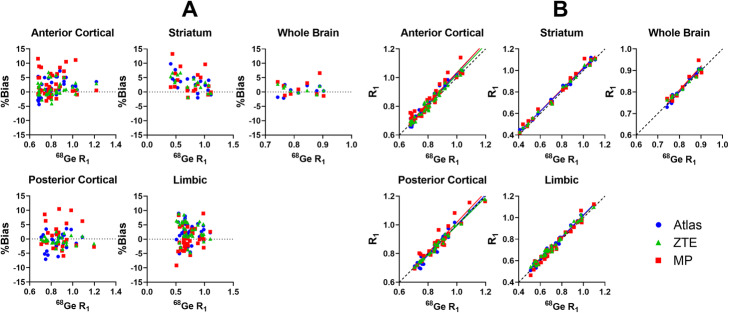
**a** Relative differences in R_1_ (% bias) plots of R_1_ when comparing Atlas-, ZTE- or MaxProb-MRAC to ^68^Ge-AC as gold standard for different clusters of brain regions. **b** Relationship between R_1_ values derived using various MRAC methods and ^68^Ge-AC as reference, where solid lines represent Deming regressions and dashed line identity

The relative bias of R_1_ estimates for various clusters of brain regions is displayed in Fig. 7a. Generally, the relative bias of R_1_ was within a range of −/+ 5%. However, for all brain clusters, the variability was greater for MaxProb-MRAC compared to the other MRAC methods. R_1_ estimates of WB had a small bias and variability for all MRAC methods. The individual regional R_1_ bias values were generally in the range of − 5 to 15%. Striatal R_1_ estimates contained mainly a positive bias, while the other regions contained both positive and negative % bias.

Strong and similar relationships were found between the R_1_ estimates from the investigated MRAC methods and the reference method (Fig. 7b). All Deming regression lines were close to the line of identity, displaying a high degree of agreement.

The outcome of the evaluation of R_1_ estimates (accuracy, precision and degree of agreement) is given for various clusters of brain regions in Table 2. For all investigated MRAC methods, the mean bias percentages were low and within a range of − 1.0 to + 3.5%. A modest negative mean bias was found for posterior cortical regions using Atlas- or ZTE-MRAC, whereas all other mean bias values were positive. Based on the SD of the mean bias percentages, ZTE-MRAC showed the highest precision and MaxProb-MRAC the lowest precision, which was consistent for most clusters of brain regions. High correlations between the evaluated MRAC methods and the reference method were attained for all brain regions (0.92–1.00).

**Table 2:** R_1_ mean % bias, precision (SD of % bias) and correlation coefficient (*r*) for all clusters of brain regions when comparing Atlas-, ZTE- and MaxProb-MRAC against ^68^Ge-MRAC. Additionally, slope and intercept of Deming regression lines are given

Brain region	MRAC	% bias	SD	r	Slope	Intercept
WB	Atlas	0.44	1.67	0.98	1.12	-0.10
	ZTE	0.99	1.21	1.00	0.94	0.06
	MaxProb	1.37	2.69	0.93	1.11	-0.08
ACR	Atlas	1.54*	2.85	0.98	1.10	-0.07
	ZTE	1.14	2.49	0.97	1.06	-0.04
	MaxProb	2.85*	4.12	0.96	1.07	-0.03
PCR	Atlas	-0.95	2.91	0.97	1.05	-0.05
	ZTE	-0.51	1.47	0.99	0.99	0.00
	MaxProb	1.14	4.38	0.92	1.06	-0.04
STR	Atlas	2.44*	2.53	0.99	0.99	0.03
	ZTE	2.71*	2.06	0.99	0.99	0.03
	MaxProb	3.17*	3.67	0.98	1.01	0.02
LR	Atlas	2.29*	3.26	0.97	1.03	-0.01
	ZTE	3.09*	3.33	0.97	1.00	0.02
	MaxProb	0.35	3.94	0.98	1.06	-0.04

*ACR* anterior cortical regions, *PCR* posterior cortical regions, *STR* striatal regions, *LR* limbic regions, *WB* whole brain

*p value < 0.05. Friedman test with post-hoc tests (comparison to ^68^Ge-AC) and Dunn’s correction

**Fig. 8:**
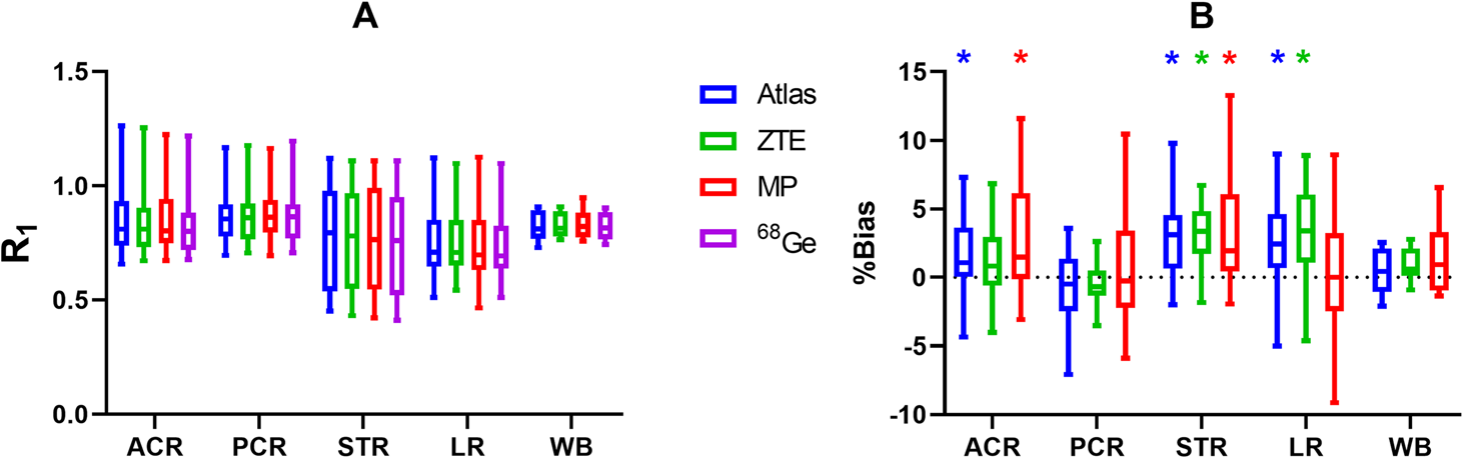
Estimated R_1_ (**a**) and % bias (**b**) using Atlas-, ZTE- or MaxProb-MRAC compared to ^68^Ge-AC for different clusters of brain regions. Bars and whiskers are median, first and third quartile. *ACR* anterior cortical regions, *PCR* posterior cortical regions, *STR* striatal regions, *LR* limbic regions, *WB* whole brain. *p value < 0.05

Boxplots for estimated R_1_ values and relative bias are given for all MRAC methods used in Fig. 8. Only minor differences in median and the variability of R_1_ estimates were found between the four MRAC methods within each brain cluster (Fig. 8a). Significant differences in relative bias were noted in the striatum for all three MRAC methods relative to ^68^Ge-MRAC, in anterior cortical regions for Atlas- and MaxProb-MRAC and in limbic regions for Atlas and ZTE, Table 2 and Fig. 8b.

### Evaluation of time-activity curves

**Fig. 9:**
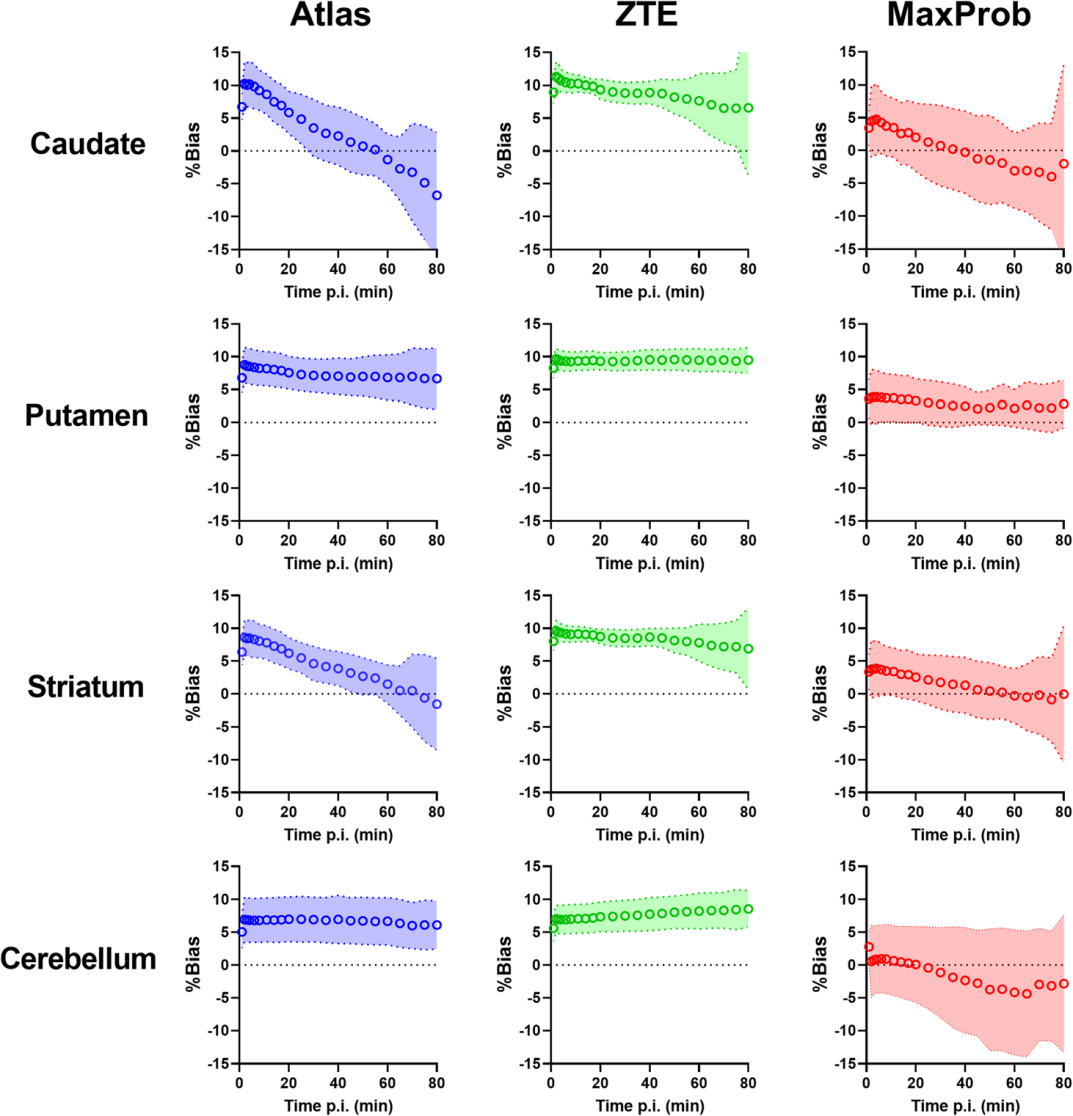
Relative bias and standard deviation on time-activity curves for striatal regions with high binding of [^11^C]PE2I (caudate nucleus, putamen and whole striatum) and cerebellum with negligible binding which was used as a reference region

Figure 9 shows the bias and its SD over time in SUV for the three evaluated MRAC methods compared to ^68^Ge-MRAC, in striatal regions and cerebellum. Particularly, in the caudate, the biases on TACs had variable shapes: a strong and slightly decreasing bias over time for ZTE-MRAC (+ 10% to7%), a relatively low bias for MaxProb-MRAC (+ 5 to − 5%) for MaxProb-MRAC and a strong and highly variable bias over time for Atlas-MRAC (+ 10 to − 7%). In cerebellum, a consistent bias over time was found for both Atlas- and ZTE-MRAC but not for MaxProb-MRAC. Intersubject variability was smallest for ZTE-MRAC and largest for MaxProb-MRAC in all considered regions.

## Discussion

### Significance

This study evaluated bias in estimated attenuation maps as well as accuracy, precision and degree of agreement of [^11^C]PE2I BP_ND_ and R_1_ estimates for three different MRAC methods using a ^68^Ge transmission scan as reference method. The main differences with most other reported MRAC evaluation studies are three-fold: (1) use of individual ^68^Ge-transmission AC maps with a direct measurement of 511 keV AC as reference method in contrast to CT-AC, (2) acquisition of dynamic data instead of static data and (3) quantification based on a kinetic modelling approach versus SUV. Reliable MRAC methods are prerequisites for both dynamic PET/MR imaging in research and clinical practice using dynamic [^11^C]PE2I PET examinations for differential diagnosis in patients with Parkinsonism [49].

### Results

*MRAC maps*—The evaluation of attenuation maps did not reveal any MRAC approach being obviously superior. ZTE- and MaxProb-MRAC agreed best with ^68^Ge-AC in brain soft tissue. In the bone, Atlas-MRAC showed the lowest and ZTE-MRAC the highest bias. While absolute differences were small in soft tissue (less than 10%), they were larger in the bone (up to 16%), with Atlas-AC showing the lowest and ZTE-MRAC the highest bias. The bias in the bone may in part be due to the spatial resolution of the different AC images, with the skull being more smoothed in the Atlas- and ^68^Ge-AC images than in the Maxprob- and ZTE-AC images. Since the bone VOI was based on ZTE, it inherently showed lower AC values in Atlas and ^68^Ge-AC maps.

For BP_ND_ in striatal regions, mean bias across AC methods ranged from about − 12 to + 9%. For R_1_, the mean bias ranged from about + 1 to + 3% in various clusters of regions across the brain for all methods. Values within limits of −/+ 5% are generally regarded as acceptable. Importantly, differences in accuracy and precision between MRAC data sets can in part be explained by differences in shapes of the time-activity curves and their SDs.

*Binding potential and relative delivery*—Based on a full quantitative analysis of the dynamic [^11^C]PE2I PET data, ZTE-MRAC showed low BP_ND_ bias for all striatal regions (− 0.9 to 1.8%). The vendor-provided Atlas-MRAC method appeared to perform well for estimation of BP_ND_ values in the putamen, but a severe and significant underestimation was found in BP_ND_ values in the caudate (12.1%). In contrast, for MaxProb-MRAC, the mean bias was also relatively low in the caudate (2.1%) while a substantial, but non-significant, bias was observed in the putamen (9.2%). For R_1_, the mean bias ranged from about + 1 to + 3% in various clusters of regions across the brain for all methods. However, MaxProb-MRAC showed higher variability in bias for both BP_ND_ and R_1_ in all regions indicating a lower precision than ZTE- and Atlas-MRAC. Methods with both a high accuracy and precision are most desirable as this will lead to more robust and reproducible outcome parameters, and this will favour ZTE-MRAC as the most appropriate MRAC in this study.

Relatively few dynamic brain PET/MRI studies have investigated the effects of MRAC methods on accuracy and precision using kinetic analysis for quantification of outcome parameters [36, 39, 43, 45, 46]. Schramm et al. [36] evaluated ZTE-MRAC and Atlas-MRAC compared to CT-based AC in eight healthy volunteers using a SIGNA PET/MRI scanner and [^18^F]PE2I. BP_ND_ in striatal regions was estimated using SRTM with cerebellum as reference region. In the striatum, the mean bias was 1.5% for ZTE-MRAC and 3.8% for Atlas-MRAC. These results are in line with our study for ZTE-MRAC, but not for Atlas-MRAC where we found a bias of − 5.4%. Our Atlas-MRAC results revealed that especially in the caudate, the BP_ND_ values were severely underestimated for all participants, leading to a range of individual biases from − 11.0 to − 1.0% compared to − 1.0 versus + 9.4 reported by Schramm et al. [36]. The discrepancy in results might be due to differences in the type of participants (patients vs healthy subjects), methodological aspects such as tracer properties ([^11^C]PE2I vs [^18^F]FE-PE2I), definition of brain regions (using Hammers maximum probability atlas in PMOD [58, 59]) and most importantly the reference standard used (^68^Ge vs CT). However, both studies point out distinctly that ZTE-MRAC performed better than Atlas-MRAC.

Merida et al. [43] assessed the value of a multi-atlas approach (MaxProb-MRAC) compared to a single atlas with CT-based AC as reference method. The study used dynamic PET data from seven healthy participants acquired on a PET/CT scanner without TOF and using the serotonin 5-HT_1A_ receptor tracer [^18^F] MPPF. SRTM was used for estimation of BP_ND_ across the brain, with cerebellar grey matter defined with the Hammers maximum probability atlas [58, 59] and excluding cerebellar vermis which may contain 5-HT_1A_ receptors. Overall, bias of BP_ND_ ranged from − 2.5 to 5.0% for MaxProb-MRAC and from − 9.3 to 3.3% for their single-atlas MRAC. For MaxProb, the range of the bias was in good agreement with our results, as was the substantial increase in bias when using a single-atlas MRAC. Furthermore, their single-atlas MRAC caused a severe underestimation of BP_ND_ values, as seen for Atlas-MRAC in our study. However, these comparisons between studies need to be interpreted with care because of differences in data acquisition, tracer kinetics and specified VOIs. It should also be noted that that study’s single-atlas MRAC implementation is different from the Atlas-MRAC in the present work.

*Evaluation of time-activity curves*—The relative bias on TACs and its SD demonstrated that the effect of the applied MRAC methods varied over time between regions and methods, especially in the caudate and consequently striatum. These findings are in line with the hypothesis that various MRAC methods are affected differently by the radioactivity distribution in the brain at different time points [43, 44, 57]. For example, for MaxProb, the bias curves for the cerebellum and striatum have similar shapes, and bias in the resulting BP_ND_ is low (2.1%). On the other hand, relative bias on TACs in the putamen and cerebellum have different shapes, resulting in a larger bias in BP_ND_ for the putamen (9.2%) despite the bias for the putamen in the TACs themselves being much lower than that of the other two methods. Similarly, for ZTE, substantial but constant biases over time were found (around 10% for the striatum and 5% for the cerebellum). These quantification errors are then compensated when using cerebellum as the reference region in kinetic modelling, and even when using SUV ratios with target region over the cerebellum. It is noteworthy that the constant-but-high biases on ZTE TACs could have even a higher impact when kinetic modelling is performed with an arterial input functions as the error compensation between two regions may not take place. In that case, MaxProb-MRAC may be the optimal method because of its lower absolute bias. Scatter correction might also contribute to the bias; however, previous reports [43, 60, 61] indicate errors produced by incorrect attenuation correction to be more significant.

*Implications*—Based on our comparisons and evaluation of the results, we suggest that the choice of MRAC method may depend on the aim of the PET examinations, tracer and quantification method. The aim of the study determines the requested accuracy and precision of the quantification method. When considering absolute values in quantitative evaluation models, MaxProb’s low absolute bias may be particularly important. For ratio or reference region-based methods, ZTE’s consistent bias over time with a low variability will be advantageous, despite a higher absolute bias compared to MaxProb-MRAC. In case of participants with an abnormal anatomy, e.g. post-trauma or post-operative patients, subjects-specific acquisition-based methods like ZTE-MRAC should be preferred. In contrast to stand-alone PET and PET/CT systems, it seems to be difficult to develop a standard MRAC routine for AC in integrated PET/MR systems suitable for all circumstances and tracers. Instead, the choice of MRAC method for a specific investigation might be based on balancing the pros and cons of each approach.

### Methodological considerations

There are some methodological considerations which need to be addressed as they might affect comparisons between MRAC methods and other studies*.* First, we used ^68^Ge-transmission AC maps with a direct voxel-wise measurement of AC, whereas CT-AC requires a conversion from Hounsfield units to 511 keV attenuation coefficients. Hence, the ^68^Ge-AC attenuation maps can be regarded as the true reference standard for AC. A comparison of ^68^Ge-AC and CT-AC demonstrated slightly higher radioactivity concentrations in PET images corrected with CT-AC: however, this increase is consistent and significant [8]. This bias should be noted when making comparisons across different imaging modalities.

In our previous study with static [^11^C]PE2I PET data [38], the effect of different AC values was investigated. The AC of 0.097 cm^−1^ measured for soft tissue for ^68^Ge-MRAC is modestly lower than that for the other investigated MRAC methods, i.e. 0.100 cm^−1^. The latter value was the same as used in clinical practice for CT-AC. In the present study, outcome parameters were normalized to the cerebellum, but we cannot exclude entirely a possible bias on BP_ND_ and R_1_ estimates due to differences in AC values.

AC of sinus regions is vulnerable to misclassification of tissue types and inaccurate registrations of templates/atlas as they are composed of the bone, soft tissue, and air [3, 32]. In theory, biased AC in sinus regions could therefore have affected the accuracy of the estimated R_1_ and BP_ND_ values in various regions. However, we believe this effect is modest. Accurate registration of templates/atlases might also be hampered by the difficulty of matching skull boundaries in sinus regions. Furthermore, the interindividual variability is large for sinus regions. Inaccurate registrations might confound parameter estimations, particularly in subcortical regions. The issues dealing with sinus segmentation have been addressed by the manufacturer [32, 62] but after the completion of the data acquisition in this work.

Like ^68^Ge-transmission AC maps, ZTE-MRAC is based on an individual scan in contrast to single- and multiple-atlas MRAC approaches. One advantage of a specific AC map for every individual is that it does not depend on a priori anatomical information and assumptions [10, 36]. Multiple atlases are expected being more reliable than a single atlas, as shown earlier by Merida et al. [43] and also found in our comparison of Atlas- and MaxProb-MRAC. However, methods based on an individual scan are likely to be suitable even for patients with an abnormal brain anatomy (e.g. brain cyst or hydrocephalus) or after surgery (e.g. patients with epilepsy surgery or traumatic brain injury).

MRI acquisitions are prone to susceptibility artefacts when the patient owns metallic implants, which could impact the performance of ZTE. However, Chiba et al. [31] reported that the magnetic susceptibility is minimal for ZTE, even at high magnetic fields (7 T MRI), due to the extremely short echo time. Hence, the risk for disturbing artefacts would be negligible. In our study, we had no patients with metallic implants as it was an exclusion criterion.

Finally, the vendor-provided ZTE- and Atlas-MRAC are under continuous development and reflected the state of the art at the time when the study was conducted. The version of ZTE-MRAC as used in the present work (MP26) is commercially available with small modifications mainly in terms of image resolution [27], but further developments have already been proposed [40] and may be implemented in forthcoming upgrades.

### Limitations

A limitation related to the design was that the results were based on data from only nine patients with Parkinsonism and this might have affected the robustness of our results. Some patients had even severe atrophy. In this case, MaxProb-MRAC may mislabel cerebral spinal fluid as the bone, which could cause a positive bias in the cerebellum as a result of inaccurate bone estimation [19], but this positive bias was not observed in our results. It might have been beneficial to study a healthy cohort in addition to the patient cohort, as effects of MRAC method and disease were entangled in the current design.

In the analysis, we had to deal with differences in axial FOV between scanners, and the MRAC maps for ^68^Ge and MaxProb were completed with corresponding information from the Atlas-AC map. Consequently, the lines of response associated with the added parts might have introduced a bias towards Atlas-MRAC, though more in regions outside than inside the brain. Head movements during the entire dynamic [^11^C]PE2I PET scan might also have affected the results as MRI sequences for AC are acquired during the first five minutes. Frame by frame motion correction was applied post reconstruction using identical transformation parameters for all four dynamic datasets, and thus, any remaining movement-induced errors should be the same for all four AC methods. However, the AC maps could be not corrected for any motion-induced errors, and the consequences might differ between AC methods.

Despite these limitations, this study provides important information on the accuracy and precision of four MRAC methods in a clinical setting with use of dynamic data.

### Future developments

For the future development of MRAC methods in dynamic brain PET, it will be important to consider diverse neurological applications in larger patient cohorts with specific tracers. The current data did not allow subgrouping based on, e.g. the magnitude of accuracy and precision, subtypes of Parkinsonian disorders. Application to more complex cases will show whether the developed MRAC methods are likely to perform well in clinical practice. For example, differential diagnosis of Parkinsonian disorders might be extremely challenging at an early stage due to the difficulty of distinguishing typical Parkinson’s disease and the atypical subtypes, and the confounding effect of comorbidity with other neurodegenerative and chronic diseases (e.g. dementia and diabetes). For this, it is essential that quantitative values are accurate and precise.

Another promising direction is the further development of an individual mapping of linear attenuation coefficients, as suggested by Visvikis et al. [3]. Like CT-AC, most of the reported MRAC methods disregarded possible heterogeneity in tissue by using AC maps with fixed values assigned to a limited number of tissue types. More detailed attenuation maps may be developed by optimizing the simultaneous acquired information from MRI and PET combined with templates/atlas and machine learning [39, 40, 42, 45, 63].

## Conclusions

ZTE-MRAC showed a high accuracy and precision for both BP_ND_ and R_1_ estimates. MaxProb-MRAC had statistically a similar accuracy, but a lower precision than ZTE-MRAC. Differences in accuracy and precision between MRAC methods were in part explained by differences in activity concentration bias over time. MaxProb-MRAC showed the lowest absolute bias over time which might be important when considering absolute activity concentration or SUV values in quantitative evaluation models. On the other hand, ZTE’s higher but consistent bias over time in target and reference regions seemed to be most advantageous for quantification using ratio or reference region-based evaluations.

## Data Availability

João M. Sousa (joao.sousa@surgsci.uu.se) is the corresponding author for the data used in this manuscript.

## Abbreviations

AC: Attenuation correction
BP_ND_: Binding potential (non-displaceable)
CT: Computer tomography
DAT: Dopamine transporter
FDG: Fluorodeoxyglucose
FOV: Field of view
MaxProb: Maximum probability algorithm
MRAC: MR-based attenuation correction
MRI: Magnetic resonance imaging
NEX: Number of excitations
OSEM: Ordered subset expectation maximisation
PET: Positron emission tomography
R_1_: Relative delivery
RPM: Receptor parametric mapping
SRTM: Simplified reference tissue model
T: Tesla
TOF: Time of flight
UTE: Ultrashort echo time
VOI: Volume of interest
ZTE: Zero echo time
3D: Three-dimensional
